# Effect of Different Pressure-Sensitive Adhesives on Performance Parameters of Matrix-Type Transdermal Delivery Systems

**DOI:** 10.3390/pharmaceutics12030209

**Published:** 2020-03-01

**Authors:** Behnam Dasht Bozorg, Ajay K. Banga

**Affiliations:** Center for Drug Delivery Research, Department of Pharmaceutical Sciences, College of Pharmacy, Mercer University, Atlanta, GA 30341, USA; behnam.dashtbozorg@live.mercer.edu

**Keywords:** transdermal delivery systems, pressure-sensitive adhesive, matrix type, lidocaine, in vitro permeation, in vitro release

## Abstract

Matrix-type transdermal delivery systems (TDS) are comprised of the drug dissolved or dispersed in a pressure-sensitive adhesive (PSA) matrix and are designed to provide a controlled delivery through the skin and into systemic circulation. PSAs can directly affect the permeation, release, and performance characteristics of the system. In this study we aimed to design and characterize transdermal delivery systems formulated with lidocaine—as the model drug—loaded in different PSAs, including silicone, polyisobutylene (PIB), and acrylate. TDS containing lidocaine at its saturation points were prepared by the solvent casting method. In vitro permeation studies across dermatomed porcine ear skin were performed using Franz diffusion cells. In vitro release studies were carried out using USP apparatus 5 (paddle over disk). The cumulative amount permeated from the acrylate was significantly higher than silicone and PIB. The acrylate TDS contained a ten times higher drug amount than silicone TDS, but the permeation flux was only two folds higher. Results also showed the release of drug does not linearly correlate to saturation, as the silicone TDS comprising of the lowest amount of drug loading, showed the highest percentage release indicating the choice of PSA affected the drug release and permeation profile.

## 1. Introduction

Transdermal delivery systems (TDSs) are designed to provide a controlled and prolonged delivery of the drug substance across the skin and into the systemic circulation [1,2]. TDS can be generally categorized into matrix-type and reservoir-type delivery systems. Matrix-type TDS contain the active ingredient(s) in a mixture of adhesives and other components [3]. TDS offer various advantages, including by-passing first-pass metabolism, extended duration of action, reduced adverse effects by maintaining the drug levels within the therapeutic window, and ease of administration and termination [4,5,6].

Among the TDS, matrix-type systems are most popular owing to their ease of formulation, high patient preference, less risk of accidental overdose, and low abuse potential. Drug in adhesive (DIA) systems are a type of matrix systems in which the drug is dissolved or dispersed in a polymeric matrix, which is layered between the backing membrane and a release liner [4,7]. In this design, pressure-sensitive adhesives (PSAs) are used as the matrix to hold the drug and control its delivery rate in addition to fulfilling their adhesion function [2,8]. Pressure-sensitive adhesives are viscoelastic materials that adhere to a substrate upon applying slight external pressure, exert strong holding force, and leave no residue after removal from a smooth surface [2,9].

Different types of PSAs used in TDSs include polyisobutylenes (PIB), silicones, and acrylic copolymers [4]. The choice of PSA is a crucial step in the early development and formulation of TDSs and is selected based on numerous factors, including solubility, stability, compatibility, and adhesion–cohesion balance [4]. Additionally, the type of PSA and interactions between the drugs and PSA can influence the flux of a drug from PSA [10,11,12]. As a result, the selection of an appropriate PSA matrix is essential in designing a TDS.

Passive diffusion is a mechanism by which drug molecules move through the stratum corneum (SC) and is governed by Fick’s Law of diffusion. Fick’s first law of diffusion states that diffusion occurs in favor of the concentration gradient [13]. With regard to the drug loading in the vehicle, the maximum skin permeation rate is obtained when the drug is at its highest thermodynamic activity, which is in a supersaturated level [14,15]. It has been shown that the maximum skin penetration rate is depended on the thermodynamic activity (saturation state) rather than the concentration value [16]. Hence, in the majority of TDSs, the drug is loaded close to the saturation solubility in order to achieve a high thermodynamic activity and a greater force for passive diffusion across the skin [17,18]

Increasing the drug loading to reach the saturation state in the TDS makes such systems unstable as there is a high probability of drug crystallization during storage [19,20,21]. Crystallization of the drug is a major challenge in the design and formulating of TDS as it makes the TDS unstable, reduces the amount of available drug, and decreases the intended flux [21,22].

The effect of the PSA matrix on drug release and permeation profile, as well as the physical characteristics of TDSs have been understudied. Hence a comparative study in this regard can help us to better understand the effect of PSA as an integral part of TDSs on performance characteristics. In this study, we aimed to investigate the effect of three adhesive matrices, including silicone, PIB, and acrylate on the performance characteristics of TDSs. Lidocaine was selected as the model drug with good passive permeation owing to its favourable physicochemical characteristics and was investigated to find the highest concentration level in each matrix that will not lead to crystallization. TDSs were prepared with the adhesive matrices loaded with lidocaine at its saturation concentration and were then further examined and compared for in vitro drug permeation and release. Physical characterization of TDS, including tack, peel adhesion, and a shear test, was also performed.

## 2. Materials and Methods

### 2.1. Materials

Lidocaine powder was purchased from Sigma-Aldrich (St. Louis, MO, USA). Acrylates copolymer (Duro-Tak 87-2287) and polyisobutylene (Duro-Tak 87-6908) were received as gift samples from Henkel (Bridgewater, NJ, USA). Silicone adhesive (BIO-PSA 7-4301) was obtained from Dow Corning^®^ (Midland, MI, USA). 3M Scotchpak™ 1022 Release Liner Fluoropolymer Coated Polyester Film and 3M Scotchpak™ 9733 Backing Polyester Film Laminate were provided by 3M Manufacturing Company (Maplewood, MN, USA). Acetonitrile, methanol, and tetrahydrofuran were of HPLC grade and obtained from Pharmco-Aaper (Shelbyville, KY, USA). Phosphate Buffered Saline (PBS), 10× Solution was purchased from Thermo Fisher Scientific (Waltham, MA, USA). Deionized (DI) water (Milli-Q^®^ Direct 8/16 System) was used. Porcine ears were provided by a local slaughterhouse (Atlanta, GA, USA).

### 2.2. Preparation of Transdermal Delivery Systems

#### 2.2.1. Drug in Adhesive Solubility and Saturation

In this study, three following adhesives were studied: acrylates copolymer (ACR), silicone (SIL), and polyisobutylene (PIB). The ACR used in our study is a non-crosslinked vinyl acetate acrylic PSA with hydroxyl functionality. Lidocaine (MW = 234.3 g/mol, log *P* = 2.4) [23] was chosen as a model drug to study the effect of the matrices on TDS performance. To determine the saturation solubility of lidocaine in each matrix, the maximum amount of drug soluble in these adhesives was measured. One gram of adhesive was accurately weighed in a glass vial, and then lidocaine powder in small increments was added. The vial was properly sealed and was mounted on a rotating mixer for slow mixing (to avoid shear stress and air bubble formation) until the added lidocaine was completely dissolved. This procedure was repeated to reach the point that the lidocaine particles were not dissolving to any further extent. Values of the highest amount of lidocaine dissolved were recorded as saturation points for the drug containing the adhesive mixtures (wet blends). This study was performed to get a general idea of the solubility of the drug in the adhesives. These solubility values cannot be considered as the saturation point since the solvent present in the wet adhesive is capable of dissolving and incorporating a higher drug amount that can result in crystallization after solvent removal.

#### 2.2.2. Crystallization Studies

Wet blends of adhesives containing lidocaine, at different percentages lower than the respective wet blend saturation point, were prepared. A small amount of the wet blend was cast on a glass microscope slide and left at room temperature for 72 h to evaporate the solvent completely. The dried adhesive film on the slides was then observed for the presence of crystals using a polarized microscope (Leica MZ6, Leica Microsystems, Wetzlar, Germany). The highest drug percentage that showed the absence of crystals indicated the achievement of a saturation point in the dried adhesives on the glass slides. TDS crystallization studies were done subsequently to verify the glass slide crystallization study results. In TDS crystallization studies, wet blends were cast on the release liner (3M Scotchpak™ 1022) and then laminated with the backing membrane (3M Scotchpak™ 9733) after drying. The prepared systems were monitored for crystal formation over one month at room temperature. The highest percentage of lidocaine that did not lead to crystallization was chosen as the saturation point (dry weight basis) for each matrix.

#### 2.2.3. Preparation of Matrix-Type TDS

The solvent casting method was used to prepare the lidocaine matrix-type TDS with different PSAs. Wet blends of PSAs loaded with the drug at the saturation point were prepared by adding lidocaine directly into the adhesive, followed by rotary mixing for 48 h to ensure homogenous mixing. 3M Scotchpak™ 1022 Release liner polyester film was fixed on a glass panel with the coated surface facing upwards. A casting knife (Tefcrom-Teflon^®^ Coated Microm film applicator by Gardco, Pompano Beach, FL, USA) was placed on the top of the liner. The wet blend was poured directly in front of the knife-blade on the release liner and a film was cast by an automatic film applicator (Byko-drive XL by BYK Instruments, Wesel, Germany), drawing down the applicator toward the lower end of the panel with a steady speed. The film applicator was equipped with a micrometer barrel to adjust film thickness. Film thickness was set to 12 mm (1 mm = 0.001 inch) for ACR and SIL PSAs and 20 mm in case of PIB to produce a 100 gsm (g/m^2^) coat weight. The casted film on the release liner (drawdown) was observed at room temperature for two minutes to observe the behavior of the film for possible shrinkage or oozing. The drawdown was then transferred into a convection oven at the temperature of 75 °C for 20 min. The temperature was set to a value that does not exceed the boiling point of any solvent present in the wet blends. On another glass panel, a 3M Scotchpak™ 9733 backing polyester film was affixed. After the oven treatment, the release liner coated with the dried film was laminated onto the backing membrane with the help of a sponge paint roller such that to avoid any entrapments of air pockets. Laminates after preparation were punched using hollow round punches of the desired size to prepare the TDS product.

#### 2.2.4. Determination of Coat Weight and Drug Content of TDS

Round-shape TDSs (area = 0.28 cm^2^) were prepared by punching the laminates for each matrix (*n* = 4) at different sites along the casting path. Systems were accurately weighed, and the weight of the equal-sized respective release liner and the backing membrane was deducted to calculate the coat weight. To determine the drug content, each system was placed in 2.0 mL of tetrahydrofuran for the adhesive to dissolve completely. The tetrahydrofuran solution was then diluted ten times with the addition of 18 mL of methanol. The mixture was further vortexed and filtered through a 0.45 µm nylon syringe filter and then analyzed using the HPLC–UV method for lidocaine content.

### 2.3. In Vitro Permeation Testing (IVPT)

#### 2.3.1. Skin Preparation

Untreated porcine ears were obtained from a local slaughterhouse and were rinsed with water to clean any debris. The dorsal side of the porcine ear was separated from the cartilage using a scalpel. Subdermal fat tissue was removed from the skin by forceps and scissors. Hair was trimmed using an electric hair clipper. Skin pieces were then dermatomed to a thickness of 500 µm to 700 µm using a Dermatome 75 mm (by Nouvag AG, Goldach, Switzerland). Dermatomed porcine ear skin was then washed with PBS (isotonic phosphate buffer saline solution containing 137 mM NaCl, 2.7 mM KCl, 8 mM Na_2_HPO_4_, and 2 mM KH_2_PO_4_ with a pH of 7.4). Skin pieces were then dried, wrapped with parafilm, placed in a resealable plastic bag, and stored at −80 °C till further use. For the experimental study, skin pieces were immersed into PBS at room temperature to thaw and then were cut into pieces with the desired size for mounting onto the Franz diffusion cells.

#### 2.3.2. Evaluation of Skin Integrity and Thickness Measurement

Prior to the permeation studies, the resistance of the skin pieces was measured to ensure the integrity of the skin barrier function. For this purpose, a waveform generator and a digital multimeter (Agilent Technologies, Santa Clara, CA, USA) connected to Ag/AgCl electrodes were used. Each skin piece was mounted on a Franz diffusion cell with both the receptor and donor chambers filled with PBS, and the skin was allowed to equilibrate for 15 min. Following equilibration, the Ag and AgCl electrodes were inserted in the receptor and donor chambers, respectively. A load resistor (*R*_L =_ 100 kΩ) was placed in series with skin, and the voltage drop across the entire circuit (*V*_O_) and skin (*V*_S_) was recorded on the multimeter. The skin resistance (*R*_S_) was calculated based on the following Equation (1) [24,25]:

R_S_ = *V*_S_*R*_L_/(*V*_O_ − *V*_S_) (1)

where *V*_O_ is 100 mV. Resistance values were calculated and reported as kΩ. The thickness of each skin piece was measured using a digital material thickness gauge (MTG-DX2 by Checkline^®^, Cedarhurst, NY, USA).

#### 2.3.3. In Vitro Skin Permeation Setup

In vitro permeation studies were performed on Franz diffusion cells. Vertical glass jacketed diffusion cells (PermeGear, Hellertown, PA, USA) with a 9 mm-diameter opening (diffusion area of 0.64 cm^2^), flat ground joint, 5 mL receptor volume, and a stir bar (600 rpm) were used. The temperature of the receptor compartment was maintained at 37 °C using a circulating water bath connected to the water jacket around the diffusion cells, providing a skin surface temperature of 32 °C. The cells were filled with 5 mL PBS to maintain the sink condition throughout the study. Three groups were defined in this study to compare the permeation profile of lidocaine for each matrix system (*n* = 4). TDSs were prepared with lidocaine at its saturation solubility in the SIL, PIB, and ACR matrices with a concentration of 2.5%, 3.5%, and 25% (*w*/*w*), respectively. For system application, skin pieces were placed on a flat surface, then the release liner was removed from the system and the TDS was applied on the skin. A glass rod was rolled over the skin pieces with gentle pressure to ensure proper adhesion of the system to the skin. Skin pieces with systems adhered to were then mounted on Franz diffusion cells, and the donor compartment was fixed using a metal clamp. Samples (300 µL) were withdrawn from the receptor chamber at 15 min, 30 min, 1, 2, 4, 6, 8, 24, 48, and 72 h, and were analyzed for drug content using an HPLC. Each aliquot was replaced with 300 µL of fresh PBS. Results were reported as mean ± SD (*n* = 4) for each test group.

### 2.4. In Vitro Release Testing (IVRT)

In vitro release studies were carried out based on the USP apparatus 5 (paddle over disk assembly) method using a Sotax dissolution tester (Sotax AT 7smart, Westborough, MA, USA). Each vessel was filled with 500 mL of 1X PBS as the dissolution media. TDSs examined in this study were prepared with lidocaine at its saturation solubility in the SIL, PIB, and ACR matrices with a concentration of 2.5%, 3.5%, and 25% (*w*/*w*), respectively. The TDS (area = 4.91 cm^2^) were adhered to a PTFE 17 mesh and were fixed on a borosilicate watch glass using PTFE clips. the watch glass–TDS–screen sandwich assembly was then placed at the bottom of the vessel, with the mesh facing upwards (*n* = 4). A distance of 25 ± 2 mm between the paddle blade and the surface of the disk assembly was maintained during the test. The temperature of the media was set at 32 ± 0.5 °C to reflect the skin temperature and the paddle speed was set at 50 rpm. The samples (1 mL) were drawn at 15 min, 30 min, 1, 2, 4, 6, 8, 24, 48, and 72 h, and were replaced by fresh media. All samples were filtered through 0.45 µm filter syringes prior to HPLC–UV analysis.

### 2.5. Quantitative Analysis

Drug content samples and in vitro permeation and release samples were quantified using a validated HPLC-UV method. The chromatographic analysis was performed on a Waters (Milford, MA, USA) Alliance 2695 Separations Module HPLC, equipped with a quaternary pump, automatic injector, and a thermostatted column compartment coupled with a photodiode array detector (Waters 996). The method was conducted using an isocratic reverse phase technique. The separation was carried out on an Agilent Eclipse Plus C18 (4.6 × 150 mm, 5 µm) column maintained at 40 °C. The mobile phase was comprised of a 15:85 acetonitrile:buffer, with 20 mM NaH_2_PO_4_ (pH = 3 adjusted with o-phosphoric acid) used as the buffer. The flow rate was set at 1 mL/min and each run-time lasted for 10 min. The lidocaine peak was achieved with the retention time of 6.1 min. The volume of injection of 5 μL was used for drug content samples, and a 20 μL injection volume was used for in vitro permeation and release samples. Matrix-matched calibration curves were used for each analysis. Peak area values were recorded at a wavelength of 192 nm. Data acquisition and processing were performed using Empower 3 software.

### 2.6. Physical Characterization of the Developed Systems

#### 2.6.1. Tack Properties

Tack is defined as the ability of an adhesive to immediately form a bond with another surface under light contact pressure [26]. The probe tack test can be used with adhesives coated on flexible backings. The standard method was developed from the Polyken Tack Tester [27]. In the probe tack test, a probe is pushed down to the point that it makes contact with the adhesive surface and then retracted at a predefined speed. The required force to break the bond after a short period of contact with the adhesive is plotted versus time [28]. The probe tack test was performed using a Texture Analyser (TA.XT Express by Texture Technologies Corp. and Stable Micro Systems, Ltd. Systems, Hamilton, MA, USA) with the following test parameters: approach speed: 0.5 mm/s; return speed: 5.0 mm/s; hold time: 10.0 s; return distance: 10.0 mm; applied force: 500 g (SIL and PIB groups) and 1000 g (ACR group); and temperature: 25 °C (*n* = 4). The cylindrical probe was of stainless steel with a diameter of 7.00 mm and a cross-section area of 154 mm^2^. Absolute positive force, positive area, and separation distance were recorded.

#### 2.6.2. Shear Adhesion

Shear adhesion evaluates the cohesion strength of a PSA matrix and its resistance to stress. In the static shear test, the required time to skid a standard area of the TDS from the adherend plate under a standard load is measured. The shear adhesion test was conducted on a ChemInstruments static shear tester (ChemInstruments Model SS-HT-8, Fairfield, OH, USA). TDS (*n* = 4) were cut (length × width: 1 inch × ½ inch) and adhered onto a stainless-steel shear panel using a 4.5-pound hand roller. The opposite end of the backing membrane was then looped through the shear test clip and taped back on itself. The assembly was then loaded into the panel holder on the shear bank at room temperature. A 1000 g weight was then hooked to the shear clip and the time for adhesive samples to fail by falling off the test surface was recorded.

#### 2.6.3. Peel Adhesion

Peel adhesion is a key attribute of TDS, which measures the force required to peel-off a path from a surface [28]. The peel adhesion force was performed using a 180-degree peel tester (ChemInstruments Model PA-1000-180, Fairfield, OH, USA). TDS (*n* = 4) were cut (length × width: 1 inch × ½ inch) and adhered onto a stainless-steel test panel using a 4.5-pound hand roller. The leading loose end of the backing membrane was fixed in the load cell grip, and the force required to detach the system of test panel with a speed of 6 inch/min was measured.

### 2.7. Data Analysis

Data were analyzed using GraphPad Prism 8 (GraphPad Software, Inc.). Statistical differences were calculated using the two-tailed Student’s *t*-test and a *p*-value of less than 0.05 was determined as a significant difference.

## 3. Results

### 3.1. Drug in Adhesive Solubility and Saturation

The saturation solubility of lidocaine in different adhesive matrices was investigated to determine the maximum possible amount of drug that can be solubilized in each matrix without the risk of it crystalizing back upon solvent removal in the preparation process or shelf-life storage. The saturation concentration of lidocaine in the adhesive (*w*/*w*% dry-weight basis) was considered as the highest concentration in which no particles or crystallization was observed. The results are shown in Table 1.

**Figure 1:**
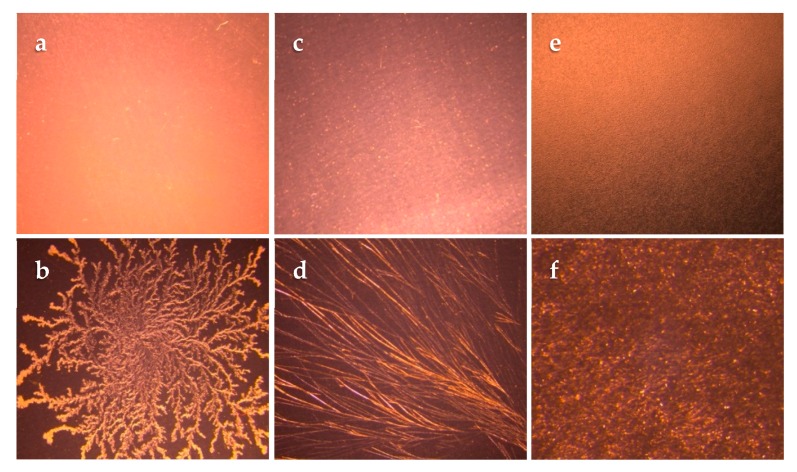
Images of prepared lidocaine transdermal delivery system (TDS) crystallization studies using polarized microscopy. Lidocaine concentration in the adhesive matrices based on the dry weight is as follows: (**a**) 2.5% (*w*/*w*) in silicone. (**b**) 3.0% (*w*/*w*) in silicone. (**c**) 25% (*w*/*w*) in acrylate. (**d**) 26% (*w*/*w*) in acrylate. (**e**) 3.5% (*w*/*w*) in PIB. (**f**) 4.0% (*w*/*w*) in PIB. (Magnification: 10×).

TDS crystallization studies revealed the saturation concentrations of lidocaine in the SIL, PIB, and ACR matrices to be 2.50, 3.50, and 25.00% (*w*/*w*), respectively. TDS at this concentration did not show any crystallization and were stable at room temperature for six months. Hence, they were further selected for permeation and release studies. Concentrations above this point resulted in the formation of crystals as shown in Figure 1. As shown in the figure, concentrations at the saturation level did not result in any crystallization when observed with polarized microscopy. However, a slight increase in the concentration values resulted in formation of crystals.

### 3.2. Coat Weight and Drug Content

Coat weight measurement and the results of drug content analysis of the prepared systems using different adhesives with lidocaine at the saturation concentration in each matrix are reported in Table 2. The results showed that all the groups (SIL, PIB, and ACR) had uniformity in both coat weight and drug content.

### 3.3. In Vitro Permeation Testing

**Figure 2:**
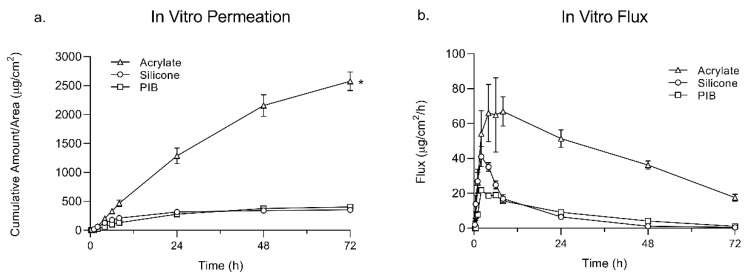
In vitro permeation (**a**) and flux (**b**) profile of lidocaine TDS comprised of lidocaine at saturation solubility in silicone, PIB, and acrylate through dermatomed porcine skin epidermis. Values are reported as mean ± SE (*n* = 4). * Represents a statistically significant difference (*p* < 0.05).

The purpose of the IVPT studies was to determine and compare the passive permeation profile of lidocaine as the model drug across the skin formulated at the saturation solubility in adhesive matrices (SIL, PIB, and ACR). The average cumulative amount of drug permeated through dermatomed porcine skin after 72 h was calculated to be 352.92 ± 63.37, 402.89 ± 16.30, and 2575.91 ± 322.14 (µg/cm^2^) for SIL, PIB, and ACR respectively. Though no difference was observed in the first hour between the three adhesives, the final amount was significantly higher in the ACR group. The permeation and flux profile of lidocaine is shown in Figure 2. The steady-state flux was calculated based on the slope of the linear portion of the cumulative amount of drug plotted versus time and X-intercept values were used to calculate the lag time. These values are reported in Table 3.

### 3.4. In Vitro Release Testing

**Figure 3:**
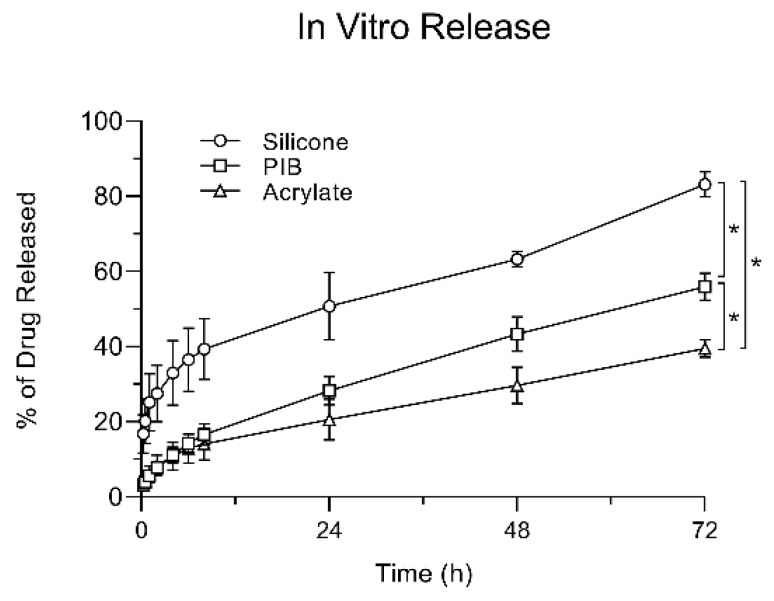
In vitro release profile of lidocaine TDS comprised of lidocaine at saturation solubility in silicone, PIB, and acrylate using the USP apparatus 5 (paddle over disk assembly). Values are presented as mean ± SE (*n* = 4). * Represents a statistically significant difference (*p* < 0.05).

The percentage of lidocaine released as a function of time is shown in Figure 3. SIL adhesive demonstrated a rapid release of the drug with significantly higher release within one hour of the study (25.23 ± 7.57), compared to ACR (6.06 ± 2.17) and PIB (5.63 ± 1.07). The total percentage released at the end of 72 h for SIL, PIB, and ACR was 83.21 ± 3.35, 55.36 ± 2.39, and 39.58 ± 3.60% respectively.

The cumulative amount of drug permeated, and the total percentage of drug released at the end of IVPT and IVRT studies are shown in Table 4 for comparison.

### 3.5. Physical Characterizations

Shear evaluation of the TDS revealed 16.88 ± 3.31, 2.38 ± 0.41, and 0.1 ± 0.00 min for SIL, PIB, and ACR, respectively, as the time required to skid the system adhered to stainless plates with a 1000 g force. In the 180° peel adhesion test, the force required to peel the system from a stainless-steel panel was measured to be 235.55 ± 35.54, 3.80 ± 1.55, and 233.83 ± 35.86 g, respectively. Although ACR showed lower shear properties and a low force was required to peel the PIB system, the TDS showed comparable tack properties with absolute positive forces of 480.33 ± 65.12, 379.70 ± 136.09, and 280.60 ± 86.80 g for the SIL, PIB, and ACR adhesives. The results of all characterization studies are summarized in Table 5.

## 4. Discussion

The effect of the pressure-sensitive adhesive (PSA) matrix was investigated on the permeation, release, and physical characteristics of TDS using SIL, PIB, and ACR matrices incorporating lidocaine at its saturation solubility.

The stratum corneum is the outermost layer of the skin, which functions as the main barrier for passive permeation allowing only small (<500 Da) and moderately lipophilic (log p of ideally about 2–3) molecules to passively permeate [5,29]. Hence lidocaine (MW = 234.3 g/mol, log *P* = 2.4) was chosen as a model drug in this study to better discriminate and understand the effect of the type of PSA on drug permeation.

While preparing a drug in the adhesive transdermal system using the solvent casting technique, determination of the saturation solubility of the drug in the adhesive is a crucial step to avoid crystallization upon evaporation of organic solvent [30]. The saturation solubility of lidocaine in the wet adhesive blend was expectedly higher when compared to slide or TDS crystallization studies since the solvent present in the wet adhesive is capable of dissolving and incorporating a higher drug amount. However, since the solvent will be removed during the TDS manufacturing process, it will cause the saturation solubility to decrease to a great extent. Slide crystallization is used as a preliminary and slightly faster technique used as an alternative to prepare TDSs for estimating the saturation solubility of drug molecules in adhesives. While the results of slide crystallization studies can provide some insight to the saturation solubility of drugs in the adhesive blend after evaporation, these values cannot be considered as the final saturation solubility as the thickness of the actual TDS, as well as processing conditions, can affect the phenomenon of crystallization [31]. Following the solubility and slide crystallization studies, the TDS crystallization studies were performed and the highest concentrations (*w*/*w*) of lidocaine in the adhesive blend in which no crystals were observed were used for further permeation, release, and crystallization studies.

To design a system with a specific coat weight value, the solid percentage (non-volatile component) of the adhesive were taken into consideration to calculate the lidocaine concentration (*w*/*w* %) on a dry-weight basis. Multiple factors can affect the coat weight, including the solid percentage of the adhesive and the thickness of the system. Since each adhesive has a different solid percentage, appropriate thicknesses were set at the casting time to reach the target coat weight value (=100 gsm). To achieve this, a higher casting thickness (20 mm) for the drug in the PIB blend was required as compared to both SIL and ACR (12 mm). Keeping the gsm values constant while preparing each adhesive system with lidocaine at its saturation level will provide information about the effect of each adhesive matrix on the lidocaine permeation and release profile.

At the end of the permeation study, the total amount of lidocaine permeated was found to be significantly higher in the ACR group compared to SIL and PIB. This can be, however, due to the higher level of drug incorporation in the ACR adhesive matrix. Interestingly, the permeation flux of lidocaine did not correlate with the drug loading. The permeation flux of lidocaine in the SIL group was higher than PIB, while it had a lower drug load. The amount of drug loading in ACR was ten times higher than SIL but it showed only a two-fold increase in the flux values. The SIL group also showed the lowest lag time while having the least amount of drug loading. This can be due to the drug-PSA intermolecular interactions, which can influence the thermodynamic activity of the drug as well as the mobility of PSA molecule, which both greatly affect skin permeation.

The results of the IVRT studies showed a similar behavior as the SIL group was releasing a higher percentage of its drug loading when compared to the other two adhesive matrices. The total percentage of drug released at the end of the study was significantly higher in the SIL group followed by PIB and then ACR, showing that drug release does not linearly correlate with the saturation point or drug loading.

There are a few studies investigating the effect of PSA matrices on drug permeation and release profiles. Liu et al. investigated the delivery of palonosetron comparing three different acrylic adhesives with no functional groups, carboxyl groups, and hydroxyl groups. Their results showed that the highest drug skin permeation amount was obtained in the acrylic adhesive with hydroxyl groups, which had a low interaction potential with the drug and high thermodynamic activity [32]. In another study performed by Park et al., the permeation rate of captopril was shown to be dependent on the type of polyacrylate copolymers [33]. Gwak et al. investigated the in vitro percutaneous absorption of ondansetron hydrochloride from the PSA matrices. Two types of ACR adhesives, one with acrylate as copolymer and the other with acrylate-vinylacetate, both with the identical functional group of carboxyl, were used. The authors demonstrated that the release rates and permeation fluxes of the drugs from the two PSAs were not significantly different from each other [34]. All these studies were more focused on the effect of different types of ACR; hence, they did not include PIB and SIL adhesives for comparison.

In another study, Kim et al. investigated the effects of various types of PSAs on the percutaneous absorption of physostigmine. The highest permeability was observed from SIL, followed by the PIB and ACR matrices. The authors discussed that the higher flux can be due to the higher thermodynamic activity of the drug in the silicone and PIB matrices in spite of the same drug content [35]. However, in our study, we designed the TDSs to maintain the same saturation level rather than the same drug load in PSA matrices. The difference in results across these studies can also be attributed to the differences in the drug’s physicochemical properties as it would make a great impact on the drug–PSA intermolecular interactions and thermodynamic activity [36].

One concerning issue regarding TDS is that after usage and upon removal of the system there is still a significant portion of the drug remaining, which not only has an abuse potential—especially in case of opioids—but also as a safety concern for the patients, family members, caregivers, and the environment [17]. The FDA Guidance for Industry—Residual Drugs in Transdermal and Related Drug Delivery Systems [37]—addresses the issue of residual drug in TDS from a safety perspective. The guidance recommends methods and appropriate scientific approaches during product development and manufacturing to minimize residual drug. TDS manufacturers are expected to make reasonable efforts to minimize this drug excess [38]. In our findings, we observed that the SIL adhesive was able to release a higher percentage of its drug loading when compared with the other two adhesive matrices. Though this cannot be generalized to other actives, such an evaluation of permeation and release at the saturation level seems to be necessary during product development to minimize the residual amount of drug in TDS. While for the selection of adhesive matrix many factors are considered, including solubility, adhesion properties, and compatibility of the adhesive matrix with the drug [39], the permeation and release profile of the active from each matrix should be taken into consideration in early development stages as we showed low solubility in a matrix does not necessarily translate into a lower flux; rather, the matrix itself plays an integral role in drug release rate regardless of loading and saturation solubility.

The results of the characterization studies indicate that the PIB systems would be easiest to remove as they required the least adhesion peel force. In the shear test, SIL systems showed more resistance to shear, indicating that such systems are less prone to show cold flow behavior when compared with the other two. All adhesives showed similar tack force, showing they have proper adhesion properties.

## 5. Conclusions

Lidocaine matrix-type transdermal delivery systems with saturation solubility in SIL, PIB, and ACR adhesives were successfully prepared and evaluated for permeation, release, and adhesion properties. Although all the TDS were prepared at saturation solubility, the choice of PSA affected the drug release and permeation profile. The ACR systems contained a ten times higher drug amount than the SIL systems, but the flux permeation was only two-fold higher. In addition, results indicate that the release of the drug does not linearly correlate to saturation, as the SIL TDS comprising of the lowest amount of drug loading, showed the highest percentage release. In this study we were able to demonstrate the understudied effect of the PSA matrix on the drug release and permeation profiles, as well as the physical characteristics of the TDSs. The results of this comparative study and similar studies can help us to better understand the effect of PSA, as an integral part of TDSs, on formulation and performance.

## Figures and Tables

**Table 1:** Saturation concentration (% *w*/*w*) of lidocaine in pressure-sensitive adhesive matrices using different techniques.

PSA	Wet Adhesive (*w*/*w* *)	Slide Crystallization (*w*/*w*)	TDS Crystallization (*w*/*w*)
Silicone	5.0%	4.5%	2.5%
Polyisobutylene	15.0%	12.5%	3.5%
Acrylate	55.0%	50.0%	25.0%

* All the concentration values were calculated based on the dry weight of the adhesive matrices.

**Table 2:** Coat weight and drug content of lidocaine matrix-type TDS at saturation point in different adhesives.

PSA	Coat Weight (g/m^2^)		Drug Content (µg/cm^2^)	
	Targeted	Experimental	Theoretical	Experimental
Silicone	100.00	95.89 ± 0.78	250.00	179.91 ± 2.52
Polyisobutylene	100.00	100.89 ± 0.82	350.00	285.27 ± 4.96
Acrylate	100.00	105.35 ± 2.66	2500.00	2786.68 ± 89.09

All experimental values are reported as mean ± SE (*n* = 4). SE: Standard error.

**Table 3:** Steady-state flux and lag time of lidocaine across dermatomed porcine skin from different adhesive matrices.

PSA	Steady-State Flux (µg/cm^2^/h)	Lag Time (h)
Silicone	32.29 ± 2.77	0.31 ± 0.09
Polyisobutylene	19.18 ± 0.87	0.52 ± 0.01
Acrylate	59.24 ± 6.21	0.54 ± 0.04

All values are reported as mean ± SE (*n* = 4). SE: Standard error.

**Table 4:** The total amount of drug permeated and the percentage of lidocaine released.

Group	Drug Content (*w*/*w*%)	Amount Permeated (µg/cm^2^)	Released (%)
Silicone	2.50	352.92 ± 63.37	83.21 ± 6.69
Polyisobutylene	3.50	402.89 ± 16.30	55.96 ± 7.20
Acrylate	25.00	2575.91 ± 322.14	39.58 ± 4.77

All values are reported as mean ± SE (*n* = 4). SE: Standard error.

**Table 5:** Results of shear, peel adhesion, and tack tests for lidocaine TDS in different adhesive matrices.

PSA	Shear Test—Time to Fail (min)	Peel Adhesion Force (g)	Tack Force (g)
Silicone	16.88 ± 3.31	235.55 ± 35.54	480.33 ± 65.12
Polyisobutylene	2.38 ± 0.41	3.80 ± 1.55	379.70 ± 136.09
Acrylate	0.10 ± 0.00	233.83 ± 35.86	280.60 ± 86.80

All values are reported as mean ± SE (*n* = 4).
